# Cerebral cortical activation and functional connectivity characteristics of children with unilateral cerebral palsy during a single-session virtual reality throwing motor task: a functional near-infrared spectroscopy study

**DOI:** 10.3389/fresc.2026.1817820

**Published:** 2026-06-02

**Authors:** Haiying Zhu, Jing Wang, Jijiang Zhou, Yanlin Wang

**Affiliations:** Department of Rehabilitation Medicine, Changzhou De'an Hospital, Changzhou, Jiangsu, China

**Keywords:** cerebral cortical activation, functional connectivity, functional near-infrared spectroscopy (fNIRS), motor-cognitive network, unilateral cerebral palsy, virtual reality motor task

## Abstract

**Objective:**

This cross-sectional study used functional near-infrared spectroscopy (fNIRS) to explore cerebral cortical activation and functional connectivity in children with Unilateral Cerebral Palsy (UCP) at resting state, during traditional non-virtual reality (non-VR) and single-session VR throwing motor tasks, aiming to clarify the differences in neural responses between VR and non-VR tasks, and provide preliminary neuroimaging evidence for VR application in pediatric cerebral palsy rehabilitation.

**Methods:**

A total of 35 children with UCP (18 males, 17 females, mean age 9.67 ± 2.4 years; 16 with left hemiplegia, 19 with right hemiplegia; 3 graded MACS Level Ⅰ, 13 Level Ⅱ, 19 Level Ⅲ) were enrolled, with the affected hemisphere standardized to the left. All participants completed fNIRS data acquisition in a randomized order under three conditions: 6-minute resting state (seated quietly with eyes closed), a single-session semi-immersive VR throwing task (8 s per movement cycle, with real-time audio-visual reward feedback for successful hits on horizontally moving virtual targets via ToF 3D motion capture), and a traditional non-VR throwing task with matched movement rhythm but no targets or feedback. fNIRS was used to monitor oxygenated hemoglobin (HbO₂) concentration in 6 regions of interest (ROIs): bilateral prefrontal (LPFC/RPFC), premotor (LPMC/RPMC) and sensorimotor cortices (LSMC/RSMC). One-way repeated measures ANOVA with FDR correction was applied for statistical analysis, which was verified by a professional biostatistician.

**Results:**

Cerebral functional connectivity strength analysis showed that after FDR correction, there were significant main effects of task condition on the FCS of LSMC-LPMC [F(2,68) = 16.956, *p* < 0.001, *η*ₚ^2^ = 0.333], LPMC-RPFC [F(2,68) = 16.256, *p* < 0.001, *η*ₚ^2^ = 0.323], and LPMC-LPFC [F(2,68) = 22.725, *p* < 0.001, *η*ₚ^2^ = 0.401], all with large effect sizes (*η*ₚ^2^ ≥ 0.14). The FCS of these ROI pairs showed a consistent trend: resting state > single-session VR throwing motor task > non-VR throwing motor task, with significant differences between any two conditions (P_FDR_ < 0.05). No statistically significant differences were observed in FCS between other ROIs (P_FDR_ > 0.05). Cerebral activation level analysis showed that after FDR correction, there were significant main effects of task condition on the activation levels of the affected-side LSMC [F(2,68) = 15.952, *p* < 0.001, *η*ₚ^2^ = 0.319] and affected-side LPFC [F(2,68) = 13.606, *p* < 0.001, *η*ₚ^2^ = 0.286], both with large effect sizes (*η*ₚ^2^ ≥ 0.14). Specifically, the activation level of LSMC followed the order: single-session VR throwing motor task > non-VR throwing motor task > resting state; the activation level of LPFC followed the order: single-session VR throwing motor task > resting state > non-VR throwing motor task, with significant differences between any two conditions (P_FDR_ < 0.05). No statistically significant differences were detected in activation levels of other brain regions (P_FDR_ > 0.05).

**Discussion:**

Based on functional near-infrared spectroscopy (fNIRS) technology, this cross-sectional study confirmed that children with unilateral cerebral palsy (UCP) exhibit distinct cerebral cortical neural activity patterns during a single-session VR throwing motor task, compared with the resting state and traditional non-VR throwing motor task. Specifically, the single-session VR motor task was associated with significantly higher activation levels in the left sensorimotor cortex (LSMC) and left lateral prefrontal cortex (LPFC) — the two regions in the affected hemisphere uniformly standardized to the left for all subjects — and modulated the functional connectivity between the premotor cortex and its functionally related brain regions. Compared with the traditional non-VR throwing motor task, the single-session VR throwing motor task elicited greater activation in the above-mentioned motor-related and higher-order cognitive brain regions, and induced a significantly different neural response pattern of the motor-cognitive network. The findings of this study reveal the cortical neural response characteristics of children with UCP during a single-session VR motor task, and provide preliminary neuroimaging evidence for subsequent studies exploring the neural mechanisms of VR-based rehabilitation in children with cerebral palsy.

## 1 Introduction

Cerebral palsy (CP) is a non-progressive syndrome characterized by motor and postural developmental disorders that occur during the brain development stage in fetuses or infants (1, 2). As a major cause of motor function impairment in children worldwide, its average prevalence is 2.11‰ (3). The pooled prevalence of cerebral palsy over the study period among 0–18 years old and different geographical regions in China was 2.07‰ (4). Approximately 80% of children with CP have comorbid upper limb dysfunction, manifested as limited range of motion, fine motor deficits, abnormal hand-eye coordination, sensory dysfunction, and decreased muscle strength, which severely affect their participation in daily activities and self-care ability (5, 6). Due to pathological changes such as structural alterations of neurons in brainstem nuclei, cortex, and gray matter masses, abnormalities of white matter nerve fibers, and myelin separation, children with CP are often accompanied by structural abnormalities including cerebral cortical atrophy, ventricular enlargement, narrowed gyri, and widened sulci (7–9). These structural changes further lead to multidimensional functional disorders such as sensory, fine motor, gross motor, and cognitive impairments (10), which not only significantly reduce the quality of life of affected children but also impose a substantial burden on family care and socioeconomic resources. Therefore, exploring scientific and efficient intervention strategies holds important clinical value and practical significance for improving the functional prognosis of children with CP and alleviating the medical burden on families and society.

Current conventional rehabilitation interventions for children with CP mainly include neurodevelopmental therapy (NDT, a widely used clinical approach in pediatric rehabilitation, though current evidence-based guidelines do not support its efficacy as a standalone intervention), hand function training, constraint-induced movement therapy, and physical agent modalities (PAMs, referring to adjunctive physical treatments including electrotherapy, thermotherapy, and ultrasound therapy) (11). However, these methods have limitations such as high repetitiveness, high training intensity, easy fatigue, and poor compliance in children (12). In recent years, virtual reality (VR) technology, with its advantages of immersion, interactivity, and imagination, can construct a multi-sensory integrated training environment, significantly enhancing children's motivation to participate in training. Studies have confirmed that positive emotional feedback in VR games can further strengthen neural stimulation (13), making it an effective alternative for upper limb function rehabilitation in CP (14, 15). Existing systematic reviews suggest that long-term VR rehabilitation training has shown potential benefits in improving upper limb motor function and promoting motor recovery in children with CP, compared with traditional non-VR rehabilitation methods (16, 17). However, the current evidence base is limited and of variable quality, and most relevant studies focus on changes in motor behavioral indicators, lacking in-depth exploration of neural activation patterns and brain functional reorganization mechanisms (18–20).

To reveal the central-level effects of VR-based motor tasks, this study introduces functional near-infrared spectroscopy (fNIRS). Based on the principle of neurovascular coupling, this technology indirectly reflects the intensity and localization of neural activity by monitoring changes in the concentrations of oxyhemoglobin (HbO₂) and deoxyhemoglobin (HbR) in the cerebral cortex (21). Compared with functional magnetic resonance imaging (fMRI), fNIRS has higher motor tolerance and environmental adaptability, does not require a closed environment or prolonged lying down, and is more suitable for children with CP who have poor cooperation (22), enabling real-time monitoring of dynamic cerebral cortical activation under conditions close to natural rehabilitation training.

This study selected brain regions closely related to upper limb motor control and sensorimotor integration as regions of interest (ROIs): 1. Bilateral lateral prefrontal cortex (LPFC/RPFC): a key brain region for high-level motor control, linking cognitive and motor functions (23); 2. Bilateral lateral premotor cortex (LPMC/RPMC): mainly responsible for movement planning, preparation and organization based on external sensory cues (24); 3. Bilateral sensorimotor cortex (LSMC/RSMC): the core executive area of the motor system, which converts motor intentions into precise actions and completes sensorimotor integration (25). The aforementioned brain regions play a core role in motor planning, execution and sensorimotor integration, and their functional reorganization is closely related to the rehabilitation efficacy of CP (26).

Existing studies have confirmed that VR environments, through high-immersion, real-time feedback, and task-oriented training scenarios, can enhance sensorimotor integration and cognitive engagement, thereby potentially inducing more robust neuroplasticity changes after long-term intervention (27, 28). However, direct neuroimaging evidence comparing cerebral cortical activation patterns and functional connectivity strength (FCS) in children with unilateral cerebral palsy (UCP) under single-session VR and non-VR motor tasks remains scarce.Based on previous neuroimaging evidence in pediatric cerebral palsy and motor control research, we defined the following *a priori* directional hypotheses before study initiation:1. For cortical activation metrics: compared with the resting state and traditional non-VR motor task, the single-session VR upper limb motor task will elicit significantly higher activation in the sensorimotor cortex and lateral prefrontal cortex (the core regions of the motor-cognitive network) in children with UCP. 2. For functional connectivity metrics: compared with the traditional non-VR motor task, the single-session VR motor task will significantly modulate the functional connectivity strength between the premotor cortex, sensorimotor cortex and prefrontal cortex, specifically, the inter-regional FCS between the above-mentioned motor-cognitive network-related ROIs will be significantly higher in the VR task state than in the non-VR task state.We hypothesized that these differences may be associated with the VR task enhancing multisensory input, improving real-time attention and motivation levels, and thus inducing a distinct task-specific neural response pattern of the motor-cognitive network. This study intends to systematically evaluate the cortical hemodynamic characteristics of children with UCP under single-session VR motor task using fNIRS technology, aiming to reveal the cortical neural response characteristics of VR motor task and provide preliminary neuroimaging reference for the optimization of clinical rehabilitation protocols for children with cerebral palsy.

## 2 Materials and methods

### 2.1 Clinical data

A total of 40 children with UCP hospitalized at Changzhou De'an Hospital from October 2023 to January 2024 were initially enrolled in this study. Five children dropped out due to failure to cooperate with the motor task, and 35 children ultimately completed the entire research process.

Inclusion Criteria: 1. Age range: 6–16 years old; 2.Meeting the diagnostic criteria for Unilateral Cerebral Palsy specified in the Guidelines for Rehabilitation of Cerebral Palsy in China (2022 Edition) (29); 3. Stable vital signs; 4. No visual or hearing impairments, capable of understanding and cooperating with task instructions; 5. Classified as Level Ⅰ∼Ⅱ on the Gross Motor Function Classification System (GMFCS); 6. Classified as ≤ Level Ⅲ on the Manual Ability Classification System (MACS) for children with cerebral palsy, i.e., having the ability to prepare or adjust movements and complete operations independently.

Exclusion Criteria: 1. Complicated with severe cardiopulmonary dysfunction, unable to tolerate the motor task or having potential cardiopulmonary safety risks related to task; 2. Poor cognitive function, unable to understand simple instructions; 3. Modified Ashworth Scale (MAS) score ≥ Grade 3, or currently using antispasmodic drugs(including benzodiazepines, baclofen, gabapentin, and botulinum toxin); 4. Having received botulinum toxin injection therapy within 6 months prior to the study; 5. Having behavioral disorders such as mental illness or severe epilepsy that hinder task implementation or cooperation.The determination of this exclusion criterion was based primarily on the subjects' previous specialist medical records, EEG results, and specialist clinical diagnosis. A pediatric rehabilitation specialist conducted a comprehensive assessment of the subjects' ability to understand study instructions, compliance with VR/non-VR tasks, and posture maintenance ability, combining clinical behavioral observation and daily rehabilitation cooperation performance, to finally exclude subjects who might interfere with fNIRS data acquisition.

Dropout Criteria: 1. Failure to complete the motor task as required by the study, affecting the completeness of data collection; 2. Contracting other diseases or developing severe complications during task period, requiring treatment or emergency rescue and thus preventing the normal progress of the research protocol.

#### 2.1.1 Structural neuroimaging data assessment

All enrolled children with UCP had routine clinical cranial computed tomography (CT) data obtained during hospital admission, which were used for the diagnostic confirmation of cerebral palsy and lateralization of cerebral lesions. However, high-resolution structural neuroimaging scans dedicated to fNIRS optode coregistration were not collected for this study. Based on routine clinical imaging reports, all subjects had structural brain lesions in the affected hemisphere consistent with the diagnosis of UCP, while individualized precise localization and quantitative assessment of lesions within our predefined 6 ROIs were not performed. The placement of fNIRS optodes was standardized in accordance with the international 10/20 electroencephalography system for population-level anatomical localization, and coregistration of optode positions to individual brain structural scans was not conducted in this study.

This study has been approved by the Medical Ethics Committee of Changzhou De'an Hospital (Ethics Approval No.: CZDALL-2021-006). The study design and implementation strictly adhere to the *World Medical Association Declaration of Helsinki* (2013 Revised Edition) and relevant medical ethics standards. Prior to the initiation of the study, trained researchers provided a detailed explanation to the legal guardians of each child regarding the study purpose, procedures, potential risks and benefits, data confidentiality measures, and the right to withdraw, etc. After the guardians fully understood and voluntarily agreed, a written informed consent form was signed. All study data were anonymized and used exclusively for the analysis of this study, with strict protection of the participants' privacy and rights.

### 2.2 Research methods

#### 2.2.1 Relevant experimental equipment

The visualized three-dimensional motion capture and analysis virtual scenario training system was the Silverfit3D system (Silverfit B.V., the Netherlands). The portable near-infrared imaging device was the Nir Smart-3000A (Danyang Huichuang Medical Equipment Co., Ltd., China).

**Figure 1:**
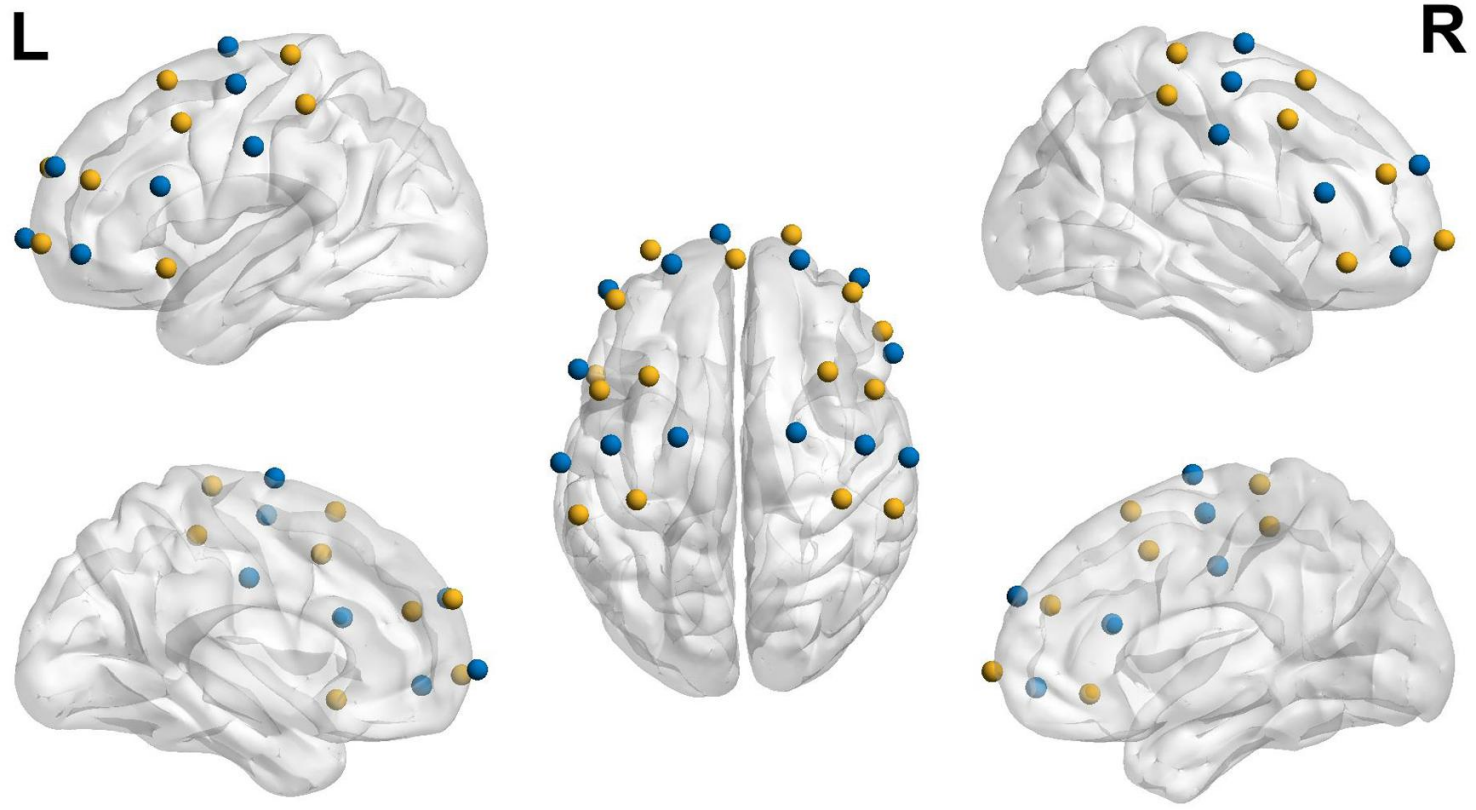
Layout of the light source and detector. Yellow solid spheres: light sources; Blue solid spheres: detectors.

**Figure 2:**
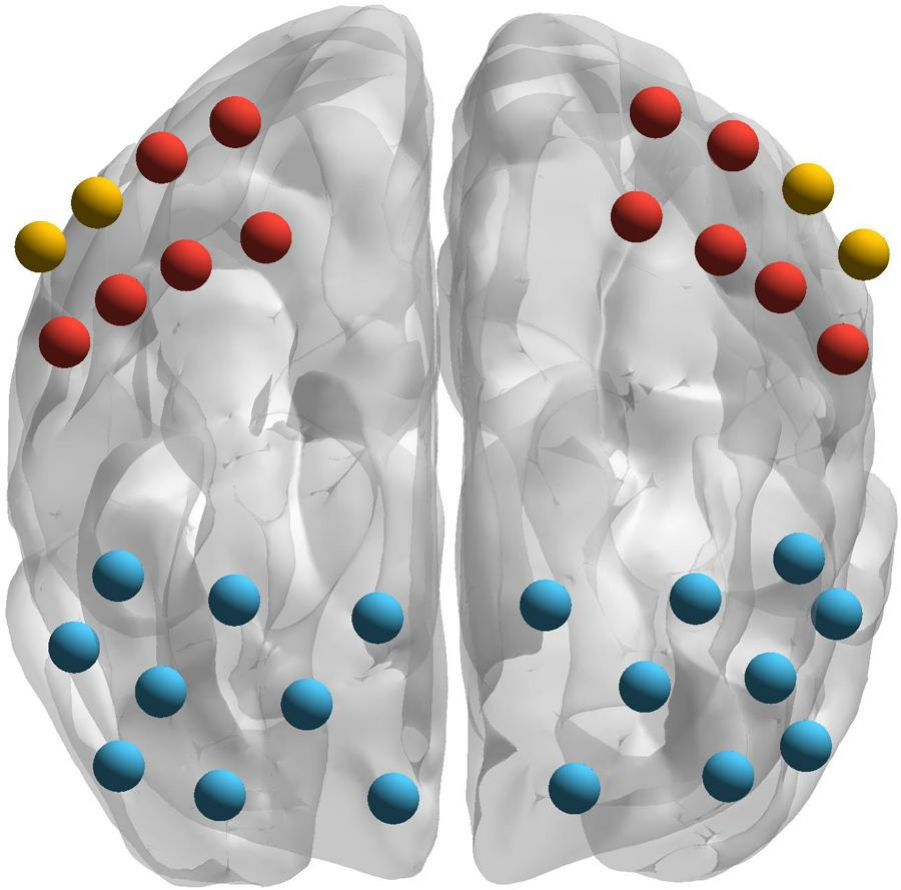
Schematic diagram of ROI brain regions. Blue: PFC; Red: PMC; Yellow: SMC.

The fNIRS system consists of a measurement cap, light source modules, detectors, and a computer host. The system operates at two wavelengths (730 nm and 850 nm) with a sampling frequency of 11 Hz. The measurement cap is equipped with 15 light sources and 13 detectors, forming 35 measurement channels with an inter-channel distance of 3 cm. The fNIRS system used in this study was combined with an electromagnetic 3D digitizer (Patriot, Polhemus Inc., Colchester, Vermont, USA), a 6 degrees of freedom (6DoF) electromagnetic 3D position tracking and digitization system developed by Polhemus Inc. (USA), which is internationally recognized as one of the gold-standard instruments for the spatial localization of optodes and electrodes in fNIRS and electroencephalography (EEG) research. The light sources and detectors of the fNIRS system were positioned in accordance with the international 10/20 EEG system standards, with Nz, A1, Cz, A2, and Iz adopted as anatomical reference points for spatial localization based on Brodmann areas (BA). In this study, we individually recorded the 3D spatial coordinates of each light source and detector using the aforementioned electromagnetic 3D digitizer, and extracted the coordinate data of the two endpoints of each measurement channel. All channels were then co-registered to the Montreal Neurological Institute (MNI) standard space and projected onto the MNI standard brain template. Based on the precise spatial coordinates of each channel, we calculated the coverage percentage of each channel across different BAs, and the brain region with the highest coverage percentage was defined as the representative functional area corresponding to the respective channel. Bilateral lateral prefrontal cortex (LPFC/RPFC, BA10); Bilateral lateral premotor cortex (LPMC/RPMC, BA6); Bilateral sensorimotor cortex (LSMC/RSMC, BA1/2/3) (30). Through spatial projection and regional mapping, the channels are divided into 6 ROIs, namely: left prefrontal cortex (LPFC), right prefrontal cortex (RPFC), left premotor cortex (LPMC), right premotor cortex (RPMC), left sensorimotor cortex (LSMC), and right sensorimotor cortex (RSMC). The probe arrangement and channel registration are shown in Figure 1*.* The specific division of ROI brain regions and the corresponding relationship with channels are presented in Table 1 and Figure 2*.*

**Table 1:** Distribution of channels in each ROI.

ROI	Channels	Brodmann Area Assignment
LPFC	8/9/10/11/21/22/23/24	BA10 (Frontopolar area)
RPFC	3/5/6/7/17/18/19/20	BA10 (Frontopolar area)
LPMC	13/14/25/26/29/30	BA6 (Pre-Motor and Supplementary Motor Cortex)
RPMC	1/2/15/16/27/28	BA6 (Pre-Motor and Supplementary Motor Cortex)
LSMC	31/32	BA1/2/3 (Primary Somatosensory Cortex)
RSMC	33/34	BA1/2/3 (Primary Somatosensory Cortex)

#### 2.2.2 Experimental procedures

To minimize errors caused by individual factors such as head circumference during data collection, a standard brain localization protocol was adopted in this study. The fNIRS measurement cap was placed on each child in a standardized and consistent manner, after which the child was seated on a chair for data acquisition. No trunk support was provided during all task procedures. Throughout the entire collection process, the room was kept dimly lit and noise-free to create stable environmental conditions for the experiment.

**Figure 3:**
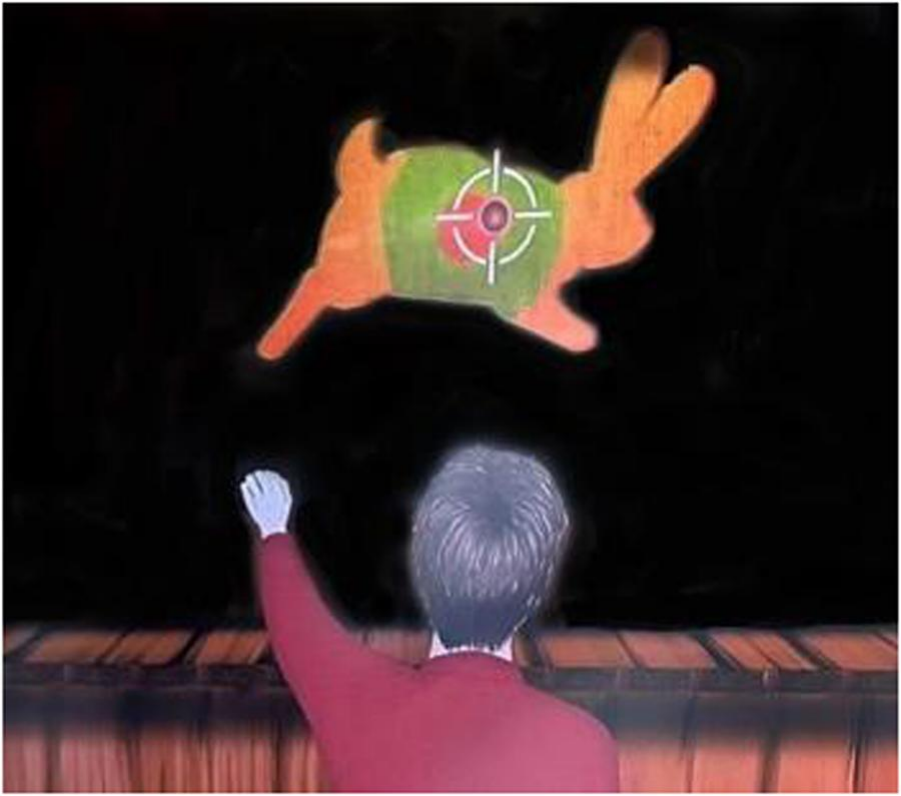
VR throwing interface.

Three data collection paradigms were used, as detailed below:

1. Resting state acquisition: Children were required to sit quietly on a chair, relax their trunk and limbs while remaining motionless, avoid head movements and speech, close their eyes, and maintain a wakeful state. Resting state data were collected continuously for 6 min.
2. Single-session VR throwing motor task: A semi-immersive virtual reality system integrated with a Time-of-Flight (ToF) 3D camera was used to capture upper limb movement details in real time at a high sampling rate via infrared sensing. Participants were seated and instructed to extend their affected upper limb to perform a throwing motion to hit horizontally moving virtual targets (wooden horses) that appeared randomly on both sides of the screen (see Figure 3).The total duration of the task data acquisition session was 6 min. The complete movement cycle of each virtual target (from appearance to disappearance) was set to 8 s, a fixed cycle designed to standardize task rhythm and unify motor load and evaluation parameters. A total of 45 target presentations and throwing attempts were completed by each participant within the 6-minute session. Notably, the entire task was performed independently and actively by all participants with no therapist assistance throughout the process.The system provided rewarding audio-visual feedback upon successful hits. The number of successful hits was automatically recorded by the system after the motor task. This movement mode was designed to induce combined shoulder and elbow joint movements, matching the core kinematic characteristics of upper limb rehabilitation training.
3. Non-VR throwing motor task: To match the kinematic core elements and time parameters of the VR task, participants were seated and performed simulated forward throwing movements of a lightweight soft ball with their affected upper limb, following the rhythm set by a metronome (one movement every 8 s). Prior to the formal task, the therapist only provided standardized verbal guidance and one-time action demonstration; no physical assistance, movement guidance, or additional prompts were provided during the entire formal task, and all movements were completed independently by participants. The non-VR task strictly matched the core kinematic elements of the VR task: movement form (combined shoulder and elbow forward throwing), movement rhythm (1 movement every 8 s), total exercise volume (45 movements in 6 min), and joint range of motion (shoulder flexion 0–90°, elbow flexion-extension 0–120°). The core differences between the two tasks are only: the VR task includes dynamic visual targets, real-time audio-visual feedback and higher cognitive load, while the non-VR task is a repetitive movement without targets or feedback. This setup ensured consistency with the VR motor task in terms of movement form, rhythm, and exercise volume, thereby effectively controlling potential confounding effects caused by differences in exercise intensity.

The random module of Python 3.12 software was used to generate 40 sets of execution sequences for the “Non-VR throwing” and “VR throwing” tasks, which were organized into a random allocation table. Researchers determined the task execution sequence for each participant according to their enrollment order with reference to this random table, to avoid the interference of sequence effects.

Prior to the formal experiment, children received 10 min of VR task operation guidance from professionally trained therapists. After confirming that the children had fully mastered the operation process, a 5-minute rest period was arranged to ensure their cerebral oxygen levels returned to the baseline state.

Formal data collection was conducted in the following order: first, 6 min of resting state data were collected, followed by data collection for the two task states (non-VR motor task and VR motor task) in the order specified in the random table. Each task state lasted for 6 min, and a 5-minute rest interval was set between each state to allow cerebral hemodynamics to return to baseline and alleviate children's fatigue and ensure data collection quality. The overall experimental workflow is shown in Table 2.

**Table 2:** Schematic diagram of the fNIRS experimental procedure.

rest	fNIRS collects resting-state data	rest	fNIRS collects VR state or non VR randomly	rest	fNIRS collects non VR state or VR state
5min	6min	5min	6min	5min	6min

### 2.3 fNIRS data analysis

NirSpark software (V1.8.9, Danyang Huichuang Medical Equipment Co., Ltd., China) was used for preprocessing of fNIRS data. To eliminate data structure heterogeneity caused by differences in the side of cerebral injury (left/right hemisphere) among children, data standardization was first performed: the left hemisphere was uniformly defined as the affected side, and the right hemisphere as the unaffected side. For data from children with an actual right-sided affected hemisphere, spatial mirror flipping of their probe placement files was conducted using the Modify SD module of NirSpark software to achieve uniform data structure.

#### 2.3.1 Data preprocessing

1. Raw fNIRS light intensity data were converted into optical density (OD) signals, and third-order cubic spline interpolation was used to correct motion artifacts (standard deviation threshold=6, absolute value threshold=0.5). A channel was marked as invalid if the standard deviation of its hemoglobin concentration time series exceeded 6 times the mean value of all valid channels. Invalid channels were excluded from subsequent analysis, and no more than 2 channels were excluded per subject; transient points with an amplitude exceeding 0.5 mM·mm were identified as motion artifacts.
2. Band-pass filtering (0.01–0.1 Hz) was applied to eliminate interference from physiological noise and drift (31).
3. The differential pathlength factor (DPF) was set to 6.0 for both wavelengths, and the filtered OD data were converted into HbO₂ and HbR concentrations using the modified Beer-Lambert law. Since previous studies have shown that HbO₂ is more sensitive to task-related changes (32), HbO₂ was selected as the primary monitoring indicator in this study.
4. Affected hemisphere standardization: For subjects with right hemiplegia, spatial mirror flipping of the probe placement file was performed using the Modify SD module of NirSpark software, and the left hemisphere was uniformly defined as the affected side.

#### 2.3.2 Calculation of functional connectivity strength and cerebral activation indicators

A steady-state time window of 30–330 s after task initiation was selected for analysis (33). This time window was designed to exclude non-steady-state physiological responses and baseline fluctuations that may occur during the initial adaptation phase of the task, ensuring that the extracted signals reflected the stable period under the task state.

FCS: Pearson correlation coefficients were calculated for the time series of oxygenated hemoglobin (*Δ*HbO₂) between each pair of channels. The correlation coefficients were converted to normally distributed z-values via Fisher r-to-z transformation, and the z-values of channels within each ROI were averaged to obtain the regional-level FCS.

Cerebral activation processing: The mean *Δ*HbO₂ concentrations of each channel were averaged to improve signal signal-to-noise ratio (SNR) and reflect the overall activation level. Subsequently, channel data were averaged by ROI to extract regional-level cerebral activation information.

### 2.4 Statistical methods

All analyses in this study were performed using SPSS 27.0 software. The entire statistical analysis protocol, assumption verification, model construction, and final results have been reviewed and verified by an independent professional biostatistician. One-way Repeated Measures Analysis of Variance (RM-ANOVA) was employed to test for within-subject differences in cerebral regional activation levels and FCS across three conditions: resting state, non-VR throwing motor task, and single-session VR throwing motor task.

For all statistical tests across multiple ROIs and ROI pairs, we controlled the family-wise Type I error rate using the Benjamini-Hochberg false discovery rate (FDR) correction, which is the most widely accepted and used method for multiple comparisons in pediatric functional neuroimaging and fNIRS studies. The correction was performed independently for two hierarchical levels of analyses, consistent with our predefined study endpoints:ROI-level activation analysis: For the one-way repeated-measures ANOVA and *post-hoc* pairwise comparisons of cortical activation levels across 3 task conditions (resting state, VR motor task, non-VR motor task) in the 6 predefined ROIs (bilateral LPFC, RPFC, LPMC, RPMC, LSMC, RSMC), FDR correction was applied to all resulting *p*-values, with the statistical significance threshold set at FDR-corrected *p* < 0.05. ROI-pair functional connectivity analysis: For the one-way repeated-measures ANOVA and *post-hoc* pairwise comparisons of functional connectivity strength across 3 task conditions in the 15 predefined ROI pairs, FDR correction was applied to all resulting *p*-values, with the statistical significance threshold set at FDR-corrected *p* < 0.05.

All statistical analyses were performed based on the *a priori* directional hypotheses defined in the Introduction section, targeting the prespecified 6 ROIs and their paired functional connectivity metrics.

RM-ANOVA was performed for core statistical analysis in this study, whose validity depends on the satisfaction of prerequisite assumptions including normality and sphericity. All relevant assumptions, alongside *a priori* sample size and statistical power verification, were confirmed prior to formal model construction, as detailed below.

The Shapiro–Wilk test was first used to verify the normality of within-subject residuals of the dependent variables for each region of interest (ROI). For dependent variables failing to meet the normality assumption (*p* < 0.05) (34), the non-parametric Friedman test was adopted as an alternative to RM-ANOVA, with *post hoc* tests corrected by the Bonferroni method. Mauchly's test of sphericity was subsequently conducted to examine whether the covariance matrix of the within-subject factor (three task conditions) satisfied the sphericity assumption. Where the test indicated a violation of the sphericity assumption (*p* < 0.05), the Greenhouse-Geisser correction was applied to adjust the degrees of freedom; where the sphericity assumption was met (*p* > 0.05), uncorrected analysis results were reported directly. For sample size and statistical power confirmation, *a priori* sample size calculation was performed using GPower 3.1 software. With the effect size partial eta-squared (*η*ₚ^2^) set to ≥ 0.14 (35), significance level *α* = 0.05, and statistical power 1-*β* = 0.80, the minimum sample size required for one-way RM-ANOVA was calculated to be 28. A total of 35 valid samples were ultimately included in this study, which exceeded the minimum requirement and met the pre-specified statistical power.

RM-ANOVA models were constructed with “task condition” as the within-subject independent variable (three levels: resting state, non-VR throwing motor task, single-session VR throwing motor task). Two sets of dependent variables were set separately: the mean *Δ*HbO₂ value of each ROI (indicator of cerebral regional activation level), and the Fisher's z-transformed value of functional connectivity strength (FCS, indicator of inter-regional functional connectivity). Core output indicators of the models included the F-value, exact numerator and denominator degrees of freedom, and *p*-value; when the sphericity assumption was violated, the Greenhouse-Geisser-corrected degrees of freedom, F-value and *p*-value were explicitly reported in the results. Partial eta-squared (*η*ₚ^2^) was calculated simultaneously to quantify the magnitude of the effect of task condition on the dependent variables, with reference to Cohen's classic effect size classification criteria for RM-ANOVA (35): *η*ₚ^2^ < 0.01 indicates a small effect, 0.01 ≤ *η*ₚ^2^ < 0.06 indicates a medium effect, and *η*ₚ^2^ ≥ 0.14 indicates a large effect. After FDR correction for multiple tests across all ROIs, a corrected *p*-value < 0.05 was considered statistically significant for the main effect of “task condition”, indicating a significant difference in the corresponding dependent variable between at least two task conditions.

Only when the FDR-corrected main effect of task condition was statistically significant, pairwise *post hoc* comparisons were performed. The Benjamini–Hochberg method was used to correct for the FDR in all pairwise comparisons. These comparisons aimed to identify the specific conditions contributing to the significant main effect and the trend of numerical changes, with a corrected P_FDR_ < 0.05 considered statistically significant. Cohen's d was calculated for all significant pairwise comparisons as the effect size indicator to quantify the magnitude of between-condition differences.

## 3 Results

### 3.1 General data

A total of 40 children were initially enrolled in accordance with the inclusion and exclusion criteria. Among them, 5 children failed to complete data collection due to inability to cooperate during the motor tasks, resulting in a final sample of 35 participants.The cohort included 18 males and 17 females, with a mean age of 9.67 ± 2.4 years. Regarding the affected side, 16 children had left hemiplegia and 19 had right hemiplegia. According to the GMFCS, 12 children were graded Level I and 23 were Level II. 2161;. Based on the MACS, 3 children were Level I, 13 were Level II, and 19 were Level III. 2162;. Their demographic characteristics and disability severity were presented in Table 3*.*

**Table 3:** Demographic and clinical characteristics of participants.

Number	Male	Female	Age (years, mean ± SD)	Left hemiplegia	Right hemiplegia	GMFCS Ⅰ	GMFCS Ⅱ	MACS Ⅰ	MACS Ⅱ	MACS Ⅲ
35	18	17	9.67 ± 2.4	16	19	12	23	3	13	19

### 3.2 Functional connectivity strength results

**Figure 4:**
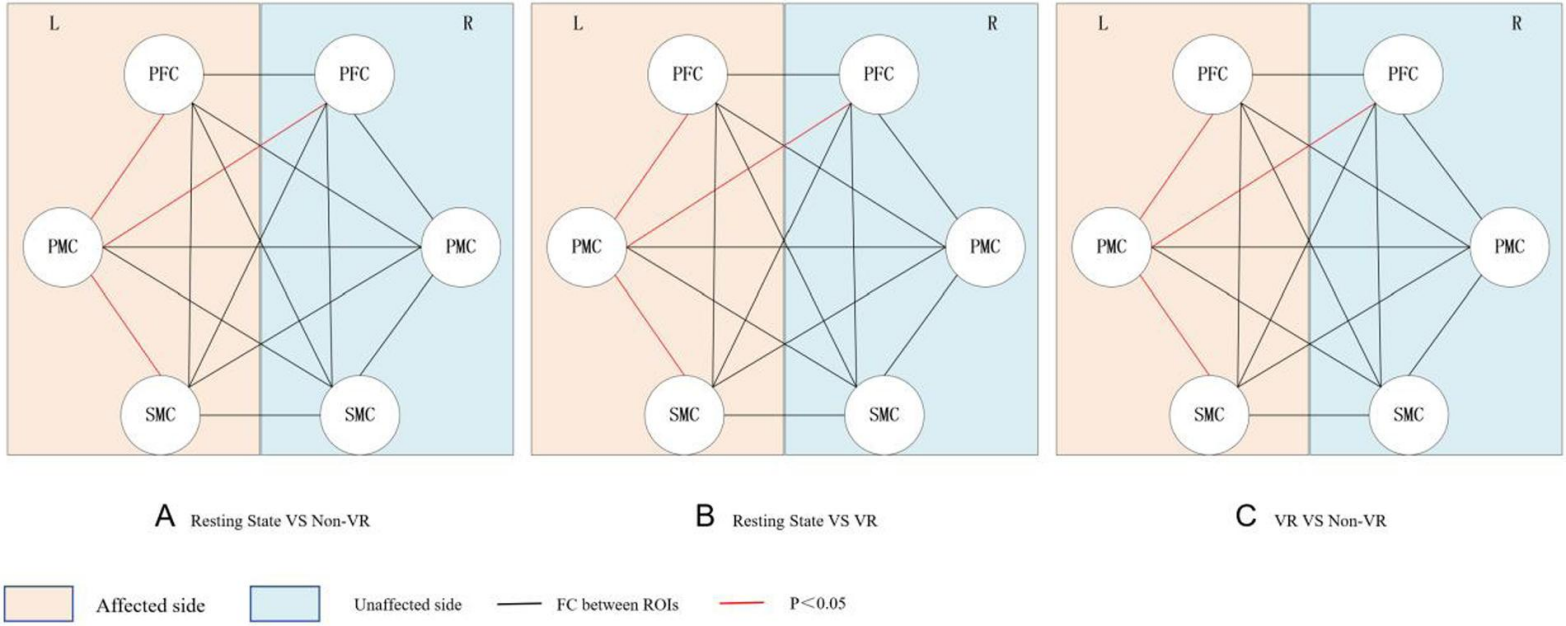
Comparison of FC strength between ROIs.

The Shapiro–Wilk test confirmed that FCS data were normally distributed for all ROI pairs (all *p* > 0.05), satisfying the normality assumption. One-way RM-ANOVA was then used to compare FCS across the three conditions: resting state, non-VR throwing motor task, and single-session VR throwing motor task. FDR correction was applied to 16 pairwise ROI tests, and Mauchly's test of sphericity was performed for each model. For models violating the sphericity assumption (*p* < 0.05), the Greenhouse-Geisser correction was applied to the degrees of freedom. Task condition had significant main effects on the FCS of LSMC-LPMC [F(2,68) = 16.96, *p* < 0.001, *η*ₚ^2^ = 0.333], LPMC-RPFC [F(2,68) = 16.26, *p* < 0.001, *η*ₚ^2^ = 0.323], and LPMC-LPFC [F(2,68) = 22.73, *p* < 0.001, *η*ₚ^2^ = 0.401] after FDR correction, all three pairs exhibited large effect sizes (*η*ₚ^2^ ≥ 0.14) (Figure 4., Table 4*.*). Pairwise *post hoc* comparisons using the Benjamini–Hochberg method revealed statistically significant differences in FCS between any two task conditions for the aforementioned ROI pairs (P_FDR_ < 0.05). The FCS of these three significant ROI pairs consistently followed the trend: resting state > single-session VR throwing motor task > non-VR throwing motor task. No significant differences in FCS were observed between other ROI pairs across the three conditions (P_FDR_ > 0.05) (Table 5*.*).

**Table 4:** Comparison of FCS between ROIs across the three paradigms.

ROI	Mauchly's Test of Sphericity	Sphericity Assumed		
	P	F	P	*η*_p_^2^
RSMC-LSMC	0.002^a^	2.109	0.129^b^	0.058
RSMC-RPMC	0.783	1.885	0.160	0.053
RSMC-LPMC	0.103	2.608	0.081	0.071
RSMC-RPFC	0.271	2.837	0.066	0.077
RSMC-LPFC	0.122	2.111	0.129	0.058
LSMC-RPMC	0.006^a^	2.804	0.068^b^	0.076
LSMC-LPMC	0.269	16.956	0.001	0.333*
LSMC-RPFC	0.045^a^	2.360	0.102^b^	0.065
LSMC-LPFC	0.133	2.251	0.113	0.062
RPMC-LPMC	0.629	2.162	0.123	0.060
RPMC-RPFC	0.298	2.210	0.118	0.061
RPMC-LPFC	0.539	2.737	0.072	0.075
LPMC-RPFC	0.001^a^	16.256	0.001^b^	0.323*
LPMC-LPFC	0.004^a^	22.725	0.001^b^	0.401*
RPFC-LPFC	0.996	1.671	0.196	0.047

*η_p_^2^ > 0.14, representing a large effect size; a: *p* < 0.05, the data did not satisfy the sphericity assumption; b: Greenhouse-Geisser corrected values were reported; All *p*-values for main effects were corrected for multiple comparisons using the Benjamini–Hochberg FDR method.

**Table 5:** Pairwise comparisons of FCS across the three paradigms.

ROI	State		Mean Difference(i-j)	SD	P_FDR_
	i	j			
LSMC-LPMC	Rest	Non VR	0.386	0.065	0.000
		VR	0.222	0.075	0.017
	Non VR	Rest	−0.386	0.065	0.000
		VR	−0.165	0.059	0.027
	VR	Rest	−0.222	0.075	0.017
		Non VR	0.165	0.059	0.027
LPMC-RPFC	Rest	Non VR	0.232	0.046	0.000
		VR	0.131	0.048	0.028
	Non VR	Rest	−0.232	0.046	0.000
		VR	−0.101	0.024	0.001
	VR	rest	−0.131	0.048	0.028
		Non VR	0.101	0.024	0.001
LPMC-LPFC	Rest	Non VR	0.301	0.055	0.000
		VR	0.212	0.048	0.000
	Non VR	Rest	−0.301	0.055	0.000
		VR	−0.089	0.032	0.029
	VR	Rest	−0.212	0.048	0.000
		Non VR	0.089	0.032	0.029

### 3.3 Cerebral activation levels results

**Figure 5:**
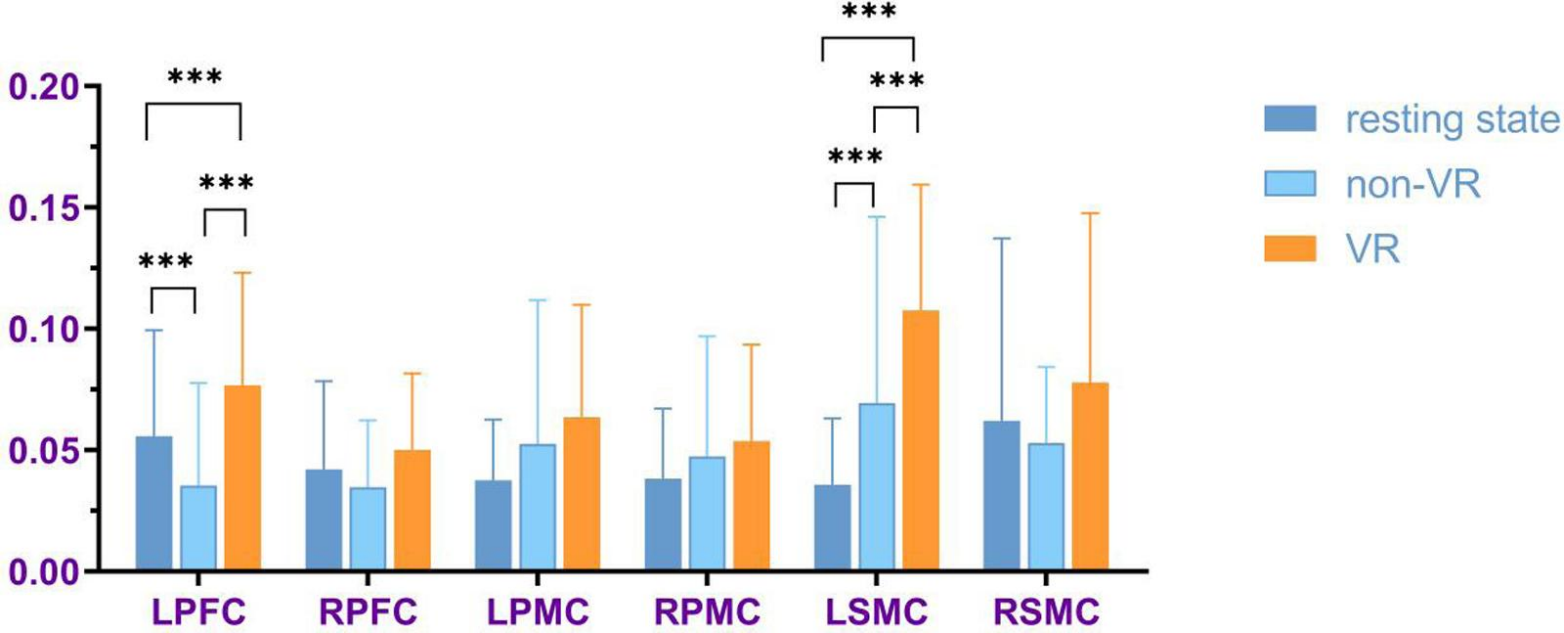
Brain functional activation map.

Using *Δ*HbO₂ concentration as the indicator of cerebral regional activation, one-way RM-ANOVA was conducted to compare activation levels across all 6 ROIs under the three task conditions. FDR correction was applied to all 6 ROI activation tests to control the overall family-wise type I error rate, and the Greenhouse-Geisser correction was applied for models violating the sphericity assumption based on Mauchly's test results. Task condition had significant main effects on the activation levels of specific brain regions after FDR correction: the affected-side LSMC [F(2,68) = 15.952, *p* < 0.001, *η*ₚ^2^ = 0.319] and the affected-side LPFC [F(2,68) = 13.606, *p* < 0.001, *η*ₚ^2^ = 0.286], both exhibiting large effect sizes (*η*ₚ^2^ ≥ 0.14). Pairwise *post hoc* comparisons using the Benjamini–Hochberg method revealed the following: For LSMC, the *Δ*HbO₂ levels showed a gradient difference in the order of single-session VR throwing motor task > non-VR throwing motor task > resting state, with statistically significant differences between all pairs (P_FDR_ < 0.05); for LPFC, the *Δ*HbO₂ levels followed the order of single-session VR throwing motor task > resting state > non-VR throwing motor task, with statistically significant differences between all pairs (P_FDR_ < 0.05). Mauchly's test of sphericity indicated a violation of the sphericity assumption for the unaffected-side RPFC (*p* = 0.025). After correction with the Greenhouse-Geisser method, the main effect of task condition on RPFC activation remained statistically non-significant [F(2,68) = 2.502, *p* = 0.123, *η*ₚ^2^ = 0.069]. No statistically significant differences in activation levels were observed for the other ROIs across the three task conditions (P_FDR_ > 0.05). Detailed statistical results of activation levels for each ROI are presented in Figure 5*.* and Tables 6, 7*.*

**Table 6:** Comparison of *Δ*HbO2 concentration across ROI.

ROI	Mauchly's Test of Sphericity	Sphericity Assumed		
	P	F	P	η_p_^2^
RSMC	0.197	1.434	0.245	0.040
LSMC	0.074	15.952	<0.001	0.319*
RPMC	0.586	2.396	0.098	0.059
LPMC	0.116	2.810	0.066	0.069
RPFC	0.025^a^	2.502^b^	0.123^b^	0.069^b^
LPFC	0.278	13.606	<0.001	0.286*

*η_p_^2^ > 0.14, representing a large effect size; a: *p* < 0.05, the data did not satisfy the sphericity assumption; b: Greenhouse-Geisser corrected values were reported; All *p*-values for main effects were corrected for multiple comparisons using the Benjamini–Hochberg FDR method.

**Table 7:** Pairwise comparisons of *Δ*HbO2 concentration across the three paradigms.

ROI	State		Mean Difference (i-j)	SD	P_FDR_
	i	j			
LSMC	Rest	VR	−0.072	0.010	0.000
		Non VR	−0.034	0.013	0.047
	VR	Rest	0.072	0.010	0.000
		Non VR	0.038	0.014	0.037
	Non VR	Rest	0.034	0.013	0.047
		VR	−0.038	0.014	0.037
LPFC	Rest	VR	−0.021	0.007	0.014
		Non VR	0.020	0.008	0.043
	Non VR	Rest	0.021	0.007	0.014
		Non VR	0.041	0.009	0.000
	Non VR	Rest	−0.020	0.008	0.043
		VR	−0.041	0.009	0.000

## 4 Discussion

Virtual reality technology has shown great potential as a novel form of feedback-based task-oriented rehabilitation intervention. Its core potential mechanism relies on constructing a realistic multi-sensory integrated scenario (integrating visual, auditory, and tactile modalities) through electronic technology, providing subjects with standardized, goal-directed motor tasks and real-time performance feedback (36). However, this study adopted a single-session VR motor task design rather than a long-term VR rehabilitation intervention. The findings of this study reflect the cerebral cortical neural response characteristics of children with UCP during a single-session VR motor task, rather than the long-term effects of VR rehabilitation training. In the single-session VR task environment, children can engage in active motor execution through human-computer interaction, which enhances the fun of the task process and improves real-time participation motivation and attention level (37). Given that this study required VR task execution under real-time monitoring with fNIRS to explore dynamic changes in cortical hemodynamics, a semi-immersive VR system was ultimately selected as the experimental platform after comprehensively considering technical compatibility and experimental safety. Although semi-immersive VR systems are slightly inferior to fully immersive VR systems in terms of visual immersion, they can minimize the risk of motion sickness in children (38, 39). Meanwhile, this system is better suited to the signal acquisition requirements of fNIRS equipment, ensuring the feasibility of experimental operations while maintaining the ecological validity of the training scenario. This provides stable and reliable experimental conditions for accurately monitoring changes in cerebral cortical hemodynamic activity during task execution. Leveraging the advantages of fNIRS technology, such as high temporal resolution and resistance to motion artifact interference, this study was able to capture and quantify in real time the dynamic evolution characteristics of cerebral cortical activation in a scenario close to clinical natural rehabilitation training, providing key technical support for revealing the cortical neural response characteristics of children with UCP during VR motor tasks.

Regarding FCS, the results of this study showed that compared with the single-session VR and non-VR throwing motor tasks, the FCS values between multiple ROIs were generally higher in the resting state. Specifically, the connectivity strengths of LSMC–LPMC, LPMC–RPFC, and LPMC–LPFC exhibited a statistically significant gradual decreasing trend across the three conditions: resting state > single-session VR throwing motor task > non-VR throwing motor task, all with large effect sizes (*η*ₚ^2^ ≥ 0.14). Existing studies have confirmed that the reduction in FCS during the task state does not indicate a downregulation of the activation level of the corresponding brain regions or a weakening of task-related neural activation (40). One potential interpretation of this finding, supported by previous neuroimaging literature, is that this FCS attenuation may reflect an adaptive neural regulatory strategy, through which the brain reduces information redundancy, optimizes neural signal transmission efficiency, and enhances information processing capacity among task-relevant neural populations (41). The attenuation of FCS when transitioning from the resting state to the task state actually reflects the brain's dynamic regulation of activity flow according to task demands, enabling the more efficient formation of task-specific activation (42). Particularly for children with cerebral palsy who have brain injuries, their baseline neural efficiency is inherently low, and additional task load can trigger the redistribution of limited neural resources, manifested as a decrease in non-essential FCS to adapt to new task requirements.

However, several alternative explanations for this finding must be considered, and the above interpretation should be treated with caution. First, the widespread FCS reduction in the task state may reflect general task-induced desynchronization of the resting-state brain network, which is a common phenomenon in functional neuroimaging studies and not necessarily associated with improved neural efficiency. Second, for children with UCP, the reduced FCS may be a compensatory manifestation of the impaired brain network's inability to maintain widespread synchronous connectivity during task execution, rather than an optimized regulatory strategy. Third, the FCS attenuation may be driven by the reallocation of attentional resources during the task, with reduced connectivity between non-core task regions as attention shifts from intrinsic resting-state cognition to external motor task execution, which is not directly linked to neural efficiency changes.

Notably, previous longitudinal intervention studies have confirmed that long-term standardized rehabilitation training can induce activity-dependent plastic changes in extensive cortical and subcortical neural tissues involved in sensory perception, motor integration, and memory formation, and this neuroplasticity is an important mechanism underlying long-term motor function recovery in children with cerebral palsy (43). However, the single-session task-related FCS changes observed in this cross-sectional study cannot be directly equated with long-term neuroplastic changes induced by rehabilitation intervention. These neural response differences can only provide a preliminary reference for exploring the potential neural mechanism of VR rehabilitation training.

Furthermore, compared with the non-VR motor task state, the single-session VR throwing motor task was associated with significantly higher connectivity strength between the LPMC in the affected hemisphere (uniformly standardized to the left hemisphere for all subjects) and the ipsilateral LSMC, bilateral LPFC and RPFC, and induced a significantly different neural response pattern of the motor-cognitive network. From the perspective of task design, the VR throwing task adopted a dynamic moving target stimulation paradigm with real-time audio-visual reward feedback, while the non-VR task was a fixed-rhythm repetitive throwing movement without external target or feedback. The former required children to dynamically adjust their movement strategies in real time based on the target's speed, direction and position. This process not only increased the demand for coordinated movement of multiple joints (shoulder, elbow, etc.) but also involved higher-order cognitive processing such as visual information decoding, movement timing judgment, and movement parameter optimization, significantly increasing the demands on both the motor system and the cognitive system. This difference in task complexity and cognitive load is the core task-related factor inducing the observed differences in brain FCS characteristics between the two tasks.

The pattern of enhanced neural connectivity observed in this single-session training is consistent in directional trend with the improvements in motor control and neuroplastic changes reported in previous long-term VR training studies. Specifically, Zhang et al. (44)pointed out that long-term VR rehabilitation training can enhance the stability and coordination of the shoulder, elbow, and wrist joints, thereby improving upper limb motor control. Aran et al. (45) further proposed that long-term VR rehabilitation intervention may promote cortical reorganization by enhancing children's sensory and cognitive processing abilities, which provides a potential theoretical reference for the neural response characteristics observed in our single-session task study. This provides a potential mechanistic explanation for the altered FCS between the PFC and motor cortices observed in this study. Studer et al. (46) emphasized that the complexity of motor tasks, required muscle strength, and speed are key factors affecting attention allocation, and the spatiotemporal judgment of dynamic targets and multi-joint coordination demands in VR significantly increase cognitive load. As the executive control center, the PFC is responsible for temporarily storing target information through working memory, retrieving motor experiences to formulate throwing strategies, and coordinating various motor brain regions to achieve goal-directed behaviors (47). Therefore, the altered FCS between the LPFC and RPFC can be regarded as a neural adaptive adjustment of the PFC in response to high cognitive demands during the VR task.

On the other hand, the PMC plays a core role in motor sequence initiation, motor control coding, and complex motor skill acquisition, particularly in integrating visual and proprioceptive information to optimize the timing and force configuration of multi-joint coordination. A study by Feng et al. (48)showed that adjusting throwing movements based on dynamic targets in VR is highly dependent on PMC function. Meanwhile, the SMC is involved in visual observation coding and multi-sensory information integration in the early stages of motor learning (49–51). The PMC and SMC synergistically regulate upper limb sensorimotor integration, spatial orientation, and joint coordination (52), collectively supporting the altered connectivity of the motor-cognitive network centered on the LPMC observed in this study.

In terms of cerebral activation, compared with the resting state, the activation levels of the bilateral PFC and right SMC were slightly lower in the non-VR motor task. It is critical to clarify that both the non-VR and VR tasks were completed independently by the participants, without any physical assistance from the therapist. This may be attributed to the fact that the non-VR task primarily involves simple, repetitive muscle movements with very low cognitive load, which triggers the redistribution of limited neural resources to the primary motor areas, resulting in decreased activation in cognitive-related brain regions. A study by Licea et al. (53) demonstrated that patients with CP exhibit decreased PFC activity during primary motor tasks, providing direct support for the findings of the present study.In contrast, the single-session VR motor task induced significantly stronger activation in the LPFC and LSMC of the affected hemisphere (uniformly standardized to the left for all subjects) compared to both the non-VR motor task and the resting state, both with large effect sizes (*η*ₚ^2^ ≥ 0.14). Surkar et al. (54)found that children with cerebral palsy show higher PFC activation during more complex motor tasks, and the degree of PFC activation is positively correlated with motor task complexity. Caruso et al. and Nunes et al. (55, 56)also pointed out that long-term VR rehabilitation training can improve attention and working memory performance in children with cerebral palsy. Compared with simple repetitive throwing, children in the single-session VR environment need to dynamically adjust their behaviors based on integrated multi-sensory stimuli, which enhances their real-time attention to movement trajectories and cognitive processing of executive strategies. This increases the cognitive load during the task and induces stronger neural activity in brain regions related to decision-making and motor planning.

Furthermore, the single-session VR motor task integrates multi-modal inputs such as visual (dynamic targets), auditory (real-time performance feedback), and proprioceptive (movement execution) information, which can induce more significant and synchronized neural activation in the sensorimotor pathways (57). This multi-modal enhanced input and feedback helps strengthen the functional coupling between the cognitive regulation of the PFC and the motor coordination of the PMC during the task. These findings reveal the differences in cortical neural response between single-session VR motor tasks and traditional simple motor tasks, and provide preliminary neuroscientific reference for subsequent research exploring VR-based rehabilitation in children with cerebral palsy.

In summary, this cross-sectional study demonstrated that, compared with the resting state and traditional non-VR throwing motor task, the single-session VR throwing motor task was associated with significant differences in FCS between the LPMC, LSMC in the affected hemisphere (uniformly standardized to the left for all subjects) and bilateral prefrontal cortices in children with UCP, while inducing significantly higher activation of the ipsilateral LPFC and LSMC, all with statistically significant large effect sizes. Compared with the non-VR motor task, the VR motor task induced a significantly different neural response pattern of the motor-cognitive network in children with UCP, which may be related to the VR environment enhancing multi-sensory input, real-time attention and participation motivation.

The core innovation of this study lies in leveraging the high temporal resolution and motion artifact tolerance advantages of fNIRS to intuitively reveal the cortical neural response characteristics of children with UCP during single-session VR motor tasks in a scenario close to natural clinical training. These findings provide preliminary neuroimaging reference for exploring the potential central neural mechanisms of VR-based rehabilitation and optimizing clinical VR rehabilitation protocols for children with cerebral palsy. However, the results of this study cannot directly prove the long-term rehabilitation efficacy of VR training, nor can they be directly equated with long-term neuroplastic changes and motor function recovery.

However, several important limitations of this study should be acknowledged, and these warrant further investigation in future research.

1. Study design limitation: This study is a cross-sectional study with a single-session task design, without a long-term VR rehabilitation intervention cohort, pre- and post-intervention behavioral assessment of motor function, and longitudinal follow-up of neuroimaging changes. Therefore, the neural response differences observed in this study cannot be directly extrapolated to long-term neuroplastic changes and motor function recovery induced by VR rehabilitation. The causal relationship between VR task-related neural activation and long-term rehabilitation efficacy needs to be further verified by large-sample randomized controlled trials (RCTs) with longitudinal follow-up.
2. Sample size and heterogeneity limitation: The sample size is relatively small, and subgroup analyses based on the side of cerebral lesion, severity of upper limb dysfunction (e.g., MACS classification), and age were not performed. This may limit the external validity of the results when generalized to children with different clinical characteristics.
3. Task design limitation: First, inherent differences exist between the resting state and two motor task conditions: resting-state data were acquired with eyes closed and no visual/motor input, while both tasks involved synchronous visual stimulation, active upper limb movement and cognitive engagement. Thus, the resting state was only suitable as a baseline for spontaneous neural activity, not for direct causal inference of task-specific neural effects. Second, the VR and non-VR tasks had inherent, incompletely matched differences in cognitive load, sensory feedback and goal-directed interaction. Although we strictly controlled the core motor load (movement form, rhythm, total volume and execution equipment) between tasks, the VR task included dynamic targets, real-time audio-visual feedback and higher cognitive engagement, while the non-VR task lacked matched feedback and interactive design. These differences introduce multiple confounds (visual input, attention, cognitive load, feedback mode and task complexity), making it impossible to specifically attribute observed neural differences to VR technology itself. In addition, the single “throwing movement” paradigm did not cover other motor modes (grasping, pointing, bimanual coordination) or gradient difficulty levels, limiting the generalizability of the conclusions to other motor scenarios.
4. Technical and neuroimaging methodology limitations: First, fNIRS technology can only detect cortical hemodynamic changes and cannot measure neural activity in subcortical structures, which are also involved in motor control and rehabilitation. Therefore, the findings of this study only reflect cortical-level neural response characteristics. Second, only routine clinical cranial structural imaging was used for the diagnostic confirmation of UCP and lesion lateralization in this study; we did not collect high-resolution individual structural brain scans for research purposes, did not perform precise localization and quantitative assessment of lesions within the predefined ROIs, and did not coregister fNIRS optode positions to individual brain scans. The presence of cortical lesions within the ROIs may affect the accuracy of fNIRS-based hemodynamic signal detection and the interpretation of neurophysiological outcomes.

Based on the above limitations, future studies can optimize the design from the following aspects:

1. Optimize study design: Introduce a large-sample RCT design, combine long-term follow-up evaluations of both behavioral indicators (e.g., MACS scores, upper limb motor function scales) and neuroimaging indicators, to systematically quantify the long-term neurobehavioral benefits of VR training and establish the causal relationship between VR-induced neural plasticity and motor function improvement.
2. Expand sample size and conduct stratified analysis: Expand the sample size and conduct stratified analyses based on the side of cerebral lesion, severity of upper limb dysfunction, and age, to explore whether the neural response characteristics to VR tasks differ among children with different clinical characteristics, and improve the external validity of the results.
3. Develop standardized and individualized VR task systems and rigorously matched control conditions: On the one hand, develop a diversified VR task system covering different motor modes (grasping, pointing, bimanual coordination, etc.) and gradient difficulty levels, and establish individualized task matching principles based on children's functional levels and rehabilitation stages. On the other hand, add a more rigorously matched control condition (e.g., 2D screen-based task with matched visual feedback, goal-directed interaction, cognitive load and task complexity) in subsequent studies, to isolate and verify the specific neural effects induced by VR technology itself, and eliminate the interference of potential confounding factors.
4. Combine high-resolution structural neuroimaging and multi-modal functional neuroimaging technologies: On the one hand, collect individual high-resolution structural brain scans to complete the coregistration of fNIRS optode positions and individual anatomical structures, and clarify the impact of lesions within predefined ROIs on fNIRS signal detection; on the other hand, combine fNIRS with other neuroimaging technologies (e.g., fMRI, EEG) to simultaneously detect cortical and subcortical neural activity, and comprehensively reveal the central neural regulatory mechanisms of VR-based rehabilitation in children with cerebral palsy.

## 5 Conclusion

Using fNIRS, this study investigated the cerebral cortical activation and functional connectivity characteristics of children with UCP under resting state, a single-session VR throwing motor task, and a matched non-VR motor task. The results showed that the VR task induced a specific neural response of the motor-cognitive network in the affected hemisphere of the children: compared with the resting state, the FCS of core brain regions related to the premotor cortex was significantly decreased under task states; compared with the non-VR task, the VR task significantly enhanced the activation of the affected LPFC and LSMC, and increased the FCS between the affected LPMC and ipsilateral sensorimotor cortex, as well as bilateral prefrontal cortices.

Our results indicate that the VR task with multisensory real-time feedback can induce adaptive neural responses in the motor-cognitive network by increasing cognitive load and engagement, highlighting the core role of the prefrontal and premotor cortices in motor control of children with UCP. Although the acute neural responses of a single-session task cannot be directly equated with the neuroplastic changes of long-term rehabilitation, this study provides preliminary neuroimaging evidence for exploring the central mechanisms of VR rehabilitation, and can offer reference for the optimization of clinical VR rehabilitation protocols for children with cerebral palsy.

## Data Availability

The original contributions presented in the study are included in the article/Supplementary Material, further inquiries can be directed to the corresponding author.
